# Using *Dictyostelium* to Develop Therapeutics for Acute Respiratory Distress Syndrome

**DOI:** 10.3389/fcell.2021.710005

**Published:** 2021-07-19

**Authors:** Sara A. Kirolos, Ramesh Rijal, Kristen M. Consalvo, Richard H. Gomer

**Affiliations:** Department of Biology, Texas A&M University, College Station, TX, United States

**Keywords:** *Dictyostelium discoideum*, chemorepulsion, acute respiratory disease syndrome, neutrophil (PMN), DPPIV, PAR2

## Abstract

Acute respiratory distress syndrome (ARDS) involves damage to lungs causing an influx of neutrophils from the blood into the lung airspaces, and the neutrophils causing further damage, which attracts more neutrophils in a vicious cycle. There are ∼190,000 cases of ARDS per year in the US, and because of the lack of therapeutics, the mortality rate is ∼40%. Repelling neutrophils out of the lung airspaces, or simply preventing neutrophil entry, is a potential therapeutic. In this minireview, we discuss how our lab noticed that a protein called AprA secreted by growing *Dictyostelium* cells functions as a repellent for *Dictyostelium* cells, causing cells to move away from a source of AprA. We then found that AprA has structural similarity to a human secreted protein called dipeptidyl peptidase IV (DPPIV), and that DPPIV is a repellent for human neutrophils. In animal models of ARDS, inhalation of DPPIV or DPPIV mimetics blocks neutrophil influx into the lungs. To move DPPIV or DPPIV mimetics into the clinic, we need to know how this repulsion works to understand possible drug interactions and side effects. Combining biochemistry and genetics in *Dictyostelium* to elucidate the AprA signal transduction pathway, followed by drug studies in human neutrophils to determine similarities and differences between neutrophil and *Dictyostelium* chemorepulsion, will hopefully lead to the safe use of DPPIV or DPPIV mimetics in the clinic.

## Acute Respiratory Distress Syndrome (ARDS)

Acute respiratory distress syndrome (ARDS) is an acute onset of low blood oxygen levels due to abnormal accumulation of multiple cell types in the lungs, including immune cells (Ashbaugh et al., 1967). The cells can damage the lungs, as well as clog airspaces, leading to lung dysfunction and thus low blood oxygen levels. The abnormal accumulation of cells is caused by either direct or indirect lung injury; direct lung injury can be from pneumonia caused by viruses, bacteria, or fungi, trauma from mechanical ventilation, or injury caused by inhaling harmful substances, while indirect lung injury is primarily from inflammation or trauma to other organ systems (Shaver and Bastarache, 2014; Cochi et al., 2016; Lynn et al., 2019). There are approximately 190,000 cases of ARDS in the United States each year (Rubenfeld et al., 2005). ARDS can have a rapid progression, with patients advancing from breathing normally despite lung damage (mild ARDS) to requiring a ventilator (moderate or severe ARDS) within a week (Ranieri et al., 2012). The mortality rate for ARDS is 27% for mild, 32% for moderate, and 45% for severe ARDS (Ranieri et al., 2012) and the 3-year mortality rate is 44, 47, and 71% respectively (Parhar et al., 2019).

**FIGURE 1:**
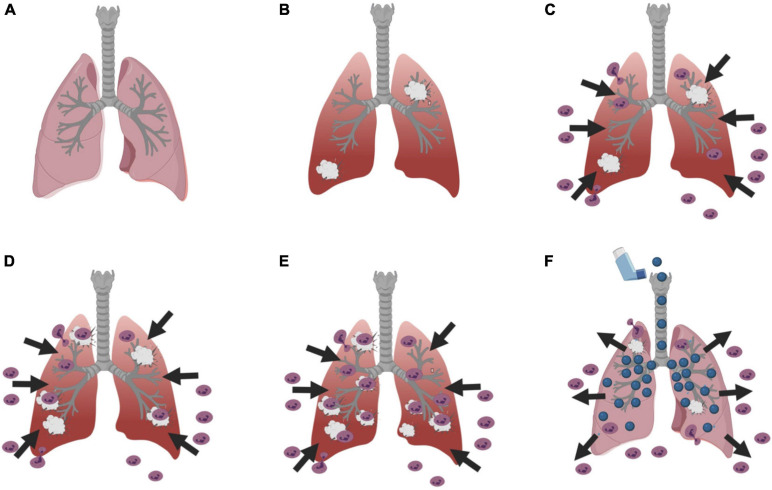
Diagram of the basic process of ARDS and a potential therapeutic mechanism. **(A)** A cartoon of a normal lung, and **(B)** a lung with damage. **(C)** Neutrophils leave the blood and enter the lung tissue and airspaces in response to the damage. **(D)** In ARDS, this can result in further lung damage, which **(E)** results in even more neutrophils attracted to the lungs causing more damage in a vicious cycle. **(F)** An intriguing possibility is that inhalation of a nebulized neutrophil chemorepellent could drive neutrophils out of the lungs and/or prevent neutrophils from entering the lungs, and thus break the vicious cycle and reduce neutrophil-induced lung damage. Figures were created using BioRender.com.

In ARDS patients, neutrophils migrate from the blood into the airspaces of the lungs (Steinberg et al., 1994; Zemans et al., 2009) in response to increased levels of inflammatory mediators, chemokines, and cell damage (Figures 1A–C; Thompson et al., 2017; Lin and Fessler, 2021). Once in the lungs, the neutrophils target pathogens for phagocytosis and release proteases, reactive oxidants, and neutrophil extracellular traps (Zemans et al., 2009). In ARDS, some neutrophils in the lungs release proteases and reactive oxygen species even if there is no pathogen present, causing lung damage, and this then recruits more neutrophils in a vicious cycle (Figures 1D,E; Weiss, 1989). The inflammation and damage reduce gas exchange, promotes vascular permeability, and increases fluid in the lung tissues and air spaces (Matthay et al., 2012; Kao et al., 2015; Lynn et al., 2019). The only effective management for ARDS is protection of the lungs, putting the patient on oxygen and a ventilator with low tidal volume ventilation to reduce stretching of the lung tissue (Acute Respiratory Distress Syndrome Network et al., 2000). There are currently no therapeutics for ARDS (Chen et al., 2020).

As described above, neutrophils entering the lungs appear to cause most of the damage in ARDS. An intriguing possible therapy for ARDS is to prevent neutrophils from entering the lungs, or to repel neutrophils that have entered the lungs back out of the lungs (Figure 1F). Some prokaryotes can sense repellent chemicals (chemorepellents) and move away from the source of the chemorepellent, and this process is fairly well understood (Pandey and Jain, 2002; Anderson et al., 2015). As described below, there are a few examples of chemorepellents in eukaryotic cells, including neutrophils, but how these affect cells is poorly understood. To move a neutrophil chemorepellent into the clinic, we need to know how this repulsion works to understand possible drug interactions and side effects.

## Chemorepulsion Can Cause Cells to Move Away From a Signal

**FIGURE 2:**
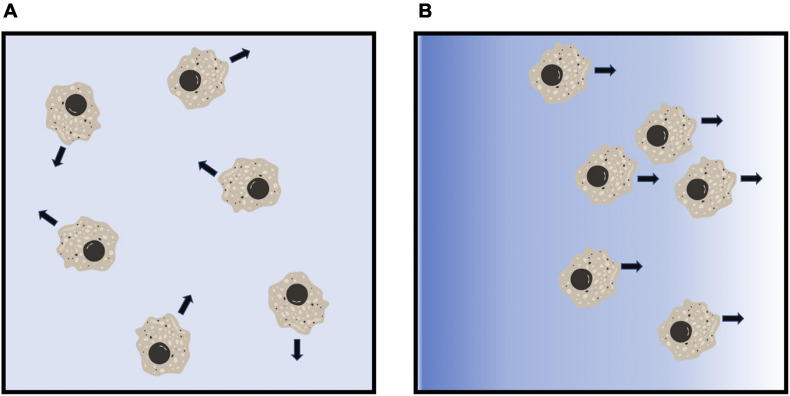
Diagram of eukaryotic chemorepulsion. **(A)** In the absence of a gradient, cells extend pseudopods, and move, in random directions. **(B)** In a gradient of a chemorepellent (blue shading), cells move away from the higher concentration of the chemorepellent. Figures were created using BioRender.com.

Chemotaxis allows migratory cells to either move toward (chemoattract) or move away (chemorepel) from an external chemotactic stimulus (Figure 2; Vianello et al., 2005). In eukaryotes, chemoattraction plays important roles during development and morphogenesis, and in immune responses (Sadik and Luster, 2012; Kolaczkowska and Kubes, 2013; Theveneau and Mayor, 2013). Directed migration of a cell toward a chemoattractant involves chemoattractant gradient sensing through a receptor-mediated signal transduction processes to induce rearrangement of cytoskeletal proteins at the front and rear of a cell, a conserved mechanism used by migrating cells (Dandekar et al., 2013; Fukujin et al., 2016). Similar to chemoattraction, chemorepulsion also plays an important role in development and immune responses (Mark et al., 1997; Vianello et al., 2005; Levine et al., 2006; Clark et al., 2014a; Guidobaldi et al., 2017; Tang, 2017). However, the mechanisms of eukaryotic chemorepulsion is still being elucidated.

The bacterium *Escherichia coli* (*E. coli*) responds to both chemoattractants and chemorepellents (Adler and Templeton, 1967; Berg and Brown, 1972; Berg and Tedesco, 1975). *E. coli* cells that sense decreasing concentrations of an attractant, or increasing concentrations of a repellent, causes the cells to tumble induced by clockwise flagellar rotation instead of smooth swimming induced by counter clockwise flagellar rotation, and the cells thus change direction to increase the probability that they will be moving in the right direction (Larsen et al., 1974). In *Helicobacter pylori*, chemorepulsion from the autocrine quorum sensing signal autoinducer-2 determines spatial organization and dispersal of biofilms (Anderson et al., 2015). The unicellular eukaryote *Trichomonas vaginalis* chemorepels from toxic agents such as metronidazole, ketoconazole, and miconazole (Sugarman and Mummaw, 1988). In the presence of a chemorepellent, such as conditioned supernatant factor (CSF), unicellular eukaryotes such as *Tetrahymena* and *Paramecium* swim backward (Rodgers et al., 2008; Plattner, 2015; Zou and Hennessey, 2017). In fungi, chemorepulsion can direct filament growth and mat formation (Karunanithi et al., 2012).

## Several Proteins Act as Neuronal Chemorepellents

In higher eukaryotes, during neuronal development, neurons extend axons to reach specific targets (Kalil et al., 2011; Santos et al., 2020). For axons to correctly navigate to their targets, attractive and repulsive factors guide neuronal growth, regeneration, and collapse (Cregg et al., 2014; Thiede-Stan and Schwab, 2015). Many of the repulsive factors inhibit growth cone outgrowth and promote their collapse (Liu et al., 2006). Some of these repulsive molecules are proteins such as Nogo, Ephrins, Semaphorins, Draxin and Netrins. Nogo-A (the active form is called Nogo-66) is expressed by many projection neurons in the central and peripheral nervous systems (Nash et al., 2009; Schwab, 2010). Ephrins are negative guidance molecules for axons (Kolpak et al., 2009; Chatzizacharias et al., 2014; Savino et al., 2015). Ephrins signal through neuronal Eph receptors including EphA5 (Wahl et al., 2000). Other repulsive molecules such as the semaphorins Sema3A and 3F bind to plexin and neuropilin co-receptors to induce the repulsion of axons (Liu et al., 2006; Andrews et al., 2013; Vo et al., 2013).

Draxin is an axon guidance cue that is vital for the development of the thick bundle of nerve fibers, called the corpus collosum, between the two hemispheres of the brain (Islam et al., 2009) and signals through the “deleted in colorectal cancer (DCC)” receptor (Islam et al., 2009; Meli et al., 2015). Although DCC is vital for axonal repulsive behavior, the binding of Netrin-1 to neuronal DCC induces attraction (Guijarro et al., 2006). Netrin-1 has both chemoattractive and chemorepulsive effects on many migratory axons during development and injury repair (Colamarino and Tessierlavigne, 1995; Furne et al., 2008). The binding of Netrin-1 to uncoordinated family member 5 (UNC-5) (Bashaw and Goodman, 1999; Hong et al., 1999) induces chemorepulsion in axons and immune cells (Hedgecock et al., 1990; Tadagavadi et al., 2010).

In mammals, multiple pathways are required for chemorepulsion (Riches et al., 2013; Holt et al., 2021). The Wnt signaling pathway is a highly conserved pathway involved in multiple cell processes such as fate determination, polarity, migration, and neural patterning (Komiya and Habas, 2008). Wnt5a activation of the non- canonical Wnt receptor RYK on specific neurons induces chemorepulsion (Clark et al., 2014b). Secreted Wnt protein also bind to Frizzled receptors, a family of integral membrane protein receptors, to repel axon growth (Huang and Klein, 2004; Freese et al., 2010). Slit guidance ligand 2 (Slit2), a chemoattractant or chemorepellent dependent on the isoform, and activation of Roundabout receptor (Robo1), a coreceptor of Slit2, induces chemorepulsion of axons as well as neutrophils (Hu, 1999; Nguyen Ba-Charvet et al., 1999; Park et al., 2016). Slit1 and Slit3 also induce chemorepulsion of olfactory tract and spinal motor axons during development (Patel et al., 2001).

## Several Proteins Act as Immune Cell Chemorepellents

In addition to Slit2, protein signals such as eotaxin-3, CXCL10, IL-8 and stromal cell derived factor-1 induce dendritic cell, monocyte, leukocyte, and/or neutrophil chemorepulsion (Ogilvie et al., 2003; Kohrgruber et al., 2004; Tharp et al., 2006). The chemokine stromal derived factor-1/CXCL12 is involved in tumor growth, metastasis and promotion of tumor immunity (Chen et al., 1994; Jager et al., 1996), possibly because CXCL12 secreted by tumors decreases T-cell infiltration (Vianello et al., 2006; d’Onofrio, 2012). In neutrophils, Slit2 plays a role in neutrophil migration toward and away from regions of inflammation (Tole et al., 2009; Ye B. Q. et al., 2010; Pilling et al., 2019). A ∼110- kDa N-terminal fragment of Slit2 induces chemorepulsion in neutrophils, and inhibiting Slit2 receptors Robo1 and syndecan-4 diminishes neutrophil chemorepulsion (Pilling et al., 2019).

## Eukaryotic Chemorepulsion Involves Regulation of the Cytoskeleton

Downstream of their receptors, many axon growth chemorepellents induce axonal repulsion by activating Rho GTPases, small signaling G protein molecular switches that play a role in cytoskeletal organization, cell movement, and cell polarity (Hall, 1998; Bustelo et al., 2007; Ridley, 2015). The activation of Rho GTPases in turn activates Rho-associated protein kinases (ROCKs), effector molecules downstream of Rho GTPases that play a role in cell shape and movement by regulating cytoskeletal elements (Leung et al., 1996; Maekawa et al., 1999). The signal transduction pathways then diverge to rearrange essential cytoskeletal proteins necessary for directed cell migration (Schmandke et al., 2007; Gelfand et al., 2009; Schwab, 2010).

## *Dictyostelium discoideum* Secretes An Endogenous Chemorepellent Called AprA

The simple eukaryote *Dictyostelium discoideum* is an excellent model system to study chemotaxis. In a nutrient-rich environment, *D. discoideum* cells grow and proliferate as single cells. When the nutrients become depleted, *D. discoideum* cells aggregate and form multicellular structures bearing spores that can survive harsh conditions (Bonner and Scharf, 1978; Kessin, 2001). The aggregation is mediated by cells secreting, and moving toward, relayed pulses of 3′, 5′-cyclic adenosine monophosphate (cAMP) (Bonner, 1970; Tomchik and Devreotes, 1981).

During their growth phase, *D. discoideum* cells secrete a protein called autocrine proliferation repressor A (AprA). AprA acts as a signal that partially inhibits cell division without inhibiting cell growth (the increase in accumulated mass) (Brock and Gomer, 2005). AprA is a 60 kDa protein which forms a ∼150 kDa complex with a protein called CfaD (Brock and Gomer, 2005; Bakthavatsalam et al., 2008).

**FIGURE 3:**
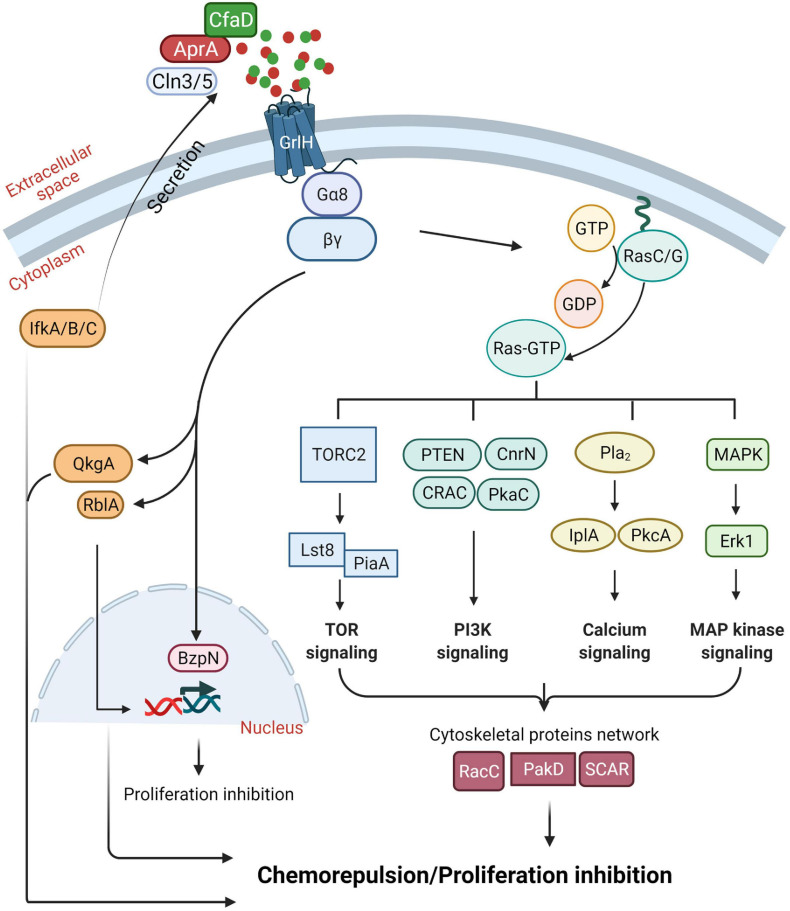
Summary of our current understanding of the AprA signal transduction pathway. See text for details. Figure created with BioRender.com.

How cells regulate the accumulation of AprA is not fully understood. Eukaryotic initiation factor 2 (eIF2) (Dever, 2002; Ye J. et al., 2010), initiation factor kinases, IfkA, IfkB, and IfkC (Fang et al., 2003; Rai et al., 2006), and Ceroid lipofuscinosis neuronal 3 (Cln3) and Cln5 that are associated with a childhood onset neurological disorder called Batten disease or neuronal ceroid lipofuscinosis (NCL) (Santavuori, 1988), are important for extracellular accumulation of AprA and CfaD (Bowman et al., 2011; Huber et al., 2014; Huber and Mathavarajah, 2018; Figure 3).

*Dictyostelium discoideum* cells lacking AprA are able to aggregate, but form abnormal structures with fewer and less viable spores, suggesting that cells lacking AprA can still migrate toward cAMP and aggregate (Brock and Gomer, 2005). In colonies of growing cells, wild type cells show directed movement away from the edge of the colony while cells lacking AprA do not move away from the edge of the colony (Phillips and Gomer, 2010). This suggested that AprA acts as a chemorepellent (Phillips and Gomer, 2010). In artificial gradients, *D. discoideum* cells move away from physiological concentrations of AprA, indicating that AprA is indeed a chemorepellent (Phillips and Gomer, 2010).

## AprA Activates a Complex Signal Transduction Pathway to Induce Chemorepulsion

*Dictyostelium discoideum* cells migrating toward cAMP and folic acid (a waste product released by bacteria, which *D. discoideum* cells uses as a chemoattractant to find and eat bacteria) require G protein-coupled receptors (GPCRs) and multiple conserved signal transduction pathways (De Wit and Bulgakov, 1985; Hadwiger et al., 1994; Ma et al., 1997; Lim et al., 2005; Kortholt et al., 2011; Yan et al., 2012). AprA activates G-proteins Gα8 and Gβ through the GPCR glutamate receptor-like H (GrlH) to induce chemorepulsion (Bakthavatsalam et al., 2009; Phillips and Gomer, 2012; Tang et al., 2018; Rijal et al., 2019; Figure 3). To decrease cell proliferation and induce chemorepulsion, AprA requires QkgA, a ROCO family kinase (Gotthardt et al., 2008; Phillips and Gomer, 2010), PakD, a member of a conserved family of p-21 activated kinases (PAKs) (Bokoch, 2003), CnrN, a phosphatase and tensin homolog (PTEN)-like phosphatase (Tang and Gomer, 2008), RblA, a homolog of a human Retinoblastoma (Rb) protein (MacWilliams et al., 2006), protein kinase A, components of the target of rapamycin (TOR) complex 2, phospholipase A, extracellular signal-regulated protein kinase (Erk1), and the Ras proteins RasC and RasG (Bakthavatsalam et al., 2009; Garcia et al., 2014; Phillips and Gomer, 2014; Tang et al., 2018; Rijal et al., 2019). In addition, AprA requires the putative bZIP transcription factor BzpN for its proliferation-inhibiting activity but not for chemorepulsion activity (Phillips et al., 2011), suggesting that AprA uses partially overlapping pathways to mediate proliferation inhibition and chemorepulsion.

## The AprA Chemorepulsion Mechanism Has a Partial but Not Complete Overlap With the camp Chemoattraction Mechanism

For chemorepulsion, in addition to the components mentioned above, AprA also uses some but not all components of the cAMP chemoattraction signal transduction pathway (Rijal et al., 2019). Similar to chemoattraction to cAMP, cells in a rAprA gradient show actin-rich protrusions at the leading edge of the cell and myosin II mediated contraction at the trailing edge of the cell, allowing the cells to move in a biased direction away from the chemorepellent (Phillips and Gomer, 2012). Although phospholipase C (PLC) and PI3 kinases 1 and 2 are important for chemorepulsion of starved *D. discoideum* cells by the synthetic cAMP analog 8-CPT-cAMP, and for cAMP chemoattraction (Keizer-Gunnink et al., 2007), PLC and PI3 kinases are not necessary for AprA chemorepulsion (Phillips and Gomer, 2012). In addition, unlike chemoattraction toward cAMP, AprA does not require Akt and PKB, guanylyl cyclases, and the cytosolic regulator of adenylate cyclase (CRAC) (Parent et al., 1998; Rijal et al., 2019).

Some of the key regulators of the AprA-induced chemorepulsion pathway are Ras GTPases. AprA induces translocation of RBDRaf1-GFP, an active Ras binding protein that when translocated to the cell cortex indicates Ras activation (Rijal et al., 2019). Although PakD is necessary for AprA mediated chemorepulsion, PakD is not necessary for AprA induced translocation of RBDRaf1-GFP to the cell cortex (Rijal et al., 2019). While PakD localizes at the rear of a migrating cell (Phillips and Gomer, 2014), PakD may negatively regulate Ras activation at the side of the cells facing away from AprA during chemorepulsion (Rijal et al., 2019). Several other proteins can negatively regulate Ras local activity at the membrane including Rho GTPases and ERK1/2 (Wang et al., 2013; Lake et al., 2016). AprA does not cause translocation of RBDRaf1-GFP in cells lacking a WASP-related cytoskeletal protein suppressor of cAR (SCAR), suggesting that AprA requires cytoskeletal proteins to activate Ras (Rijal et al., 2019).

## AprA Induces Chemorepulsion by Inhibiting Pseudopod Formation at the Side of the Cell Closest to the Source of AprA

When cells such as *D. discoideum* and neutrophils move, they induce a localized polymerization of actin to form a pseudopod that protrudes from the cell, and when this attaches to a substrate, the cells can then move following their pseudopod (King and Insall, 2009). In the absence of a chemoattractant or chemorepellent gradient, cells form pseudopods at random locations, and thus move in random directions (Sasaki et al., 2004; Rijal et al., 2019). During chemoattraction, cells polymerize actin and extend a pseudopod toward the source of the attractant (Sasaki et al., 2004). During chemorepulsion, AprA inhibits filamentous actin (F-actin) polymerization at the region of the cell closest to the source of AprA, inhibiting pseudopod formation in the sector of the cell closest to the source of AprA (Rijal et al., 2019). This then allows the cells to move in any direction except the direction toward the source of AprA, resulting in chemorepulsion. This then indicates a fundamental difference between chemoattraction and chemorepulsion in *D. discoideum*: if a chemoattractant is coming from the West, a cell will tend to extend a pseudopod and move toward the West; to a first approximation, if a chemorepellent is coming from the West, cells will extend pseudopods randomly, and move toward, the East, North, or South.

## The Identification of AprA Led to the Identification of a Human Neutrophil Chemorepellent

*Dictyostelium discoideum* and neutrophils share chemotaxis properties (Wang, 2009; Wang et al., 2011). Although AprA has little similarity to mammalian proteins, a predicted tertiary structure of AprA showed similarity to the structure of human dipeptidyl peptidase IV (DPPIV) (Zhang, 2008; Roy et al., 2010, 2012; Herlihy et al., 2013). DPPIV is a 110 kDa serine protease that localizes on the extracellular surface of some lymphocytes, endothelial cells, and is also present in plasma, serum, cerebrospinal fluid, synovial fluid, semen, and urine in a soluble form, and cleaves peptides with a proline or alanine in the second position at the N-terminus end (Walborg et al., 1985; Thoma et al., 2003; Cordero et al., 2009; Kotacková et al., 2009; Nauck et al., 2011; Pan et al., 2021). In addition to the predicted structural similarity, AprA has a DPPIV -like protease activity (Herlihy et al., 2013, 2017). Although AprA is not able to repel human neutrophils, DPPIV induces chemorepulsion of *D. discoideum* cells, suggesting a conserved mechanism of action (Herlihy et al., 2017). In a range of concentrations encompassing its concentration in human plasma, DPPIV acts as a chemorepellent for human and mouse neutrophils, but does not affect the motility of macrophages and lymphocytes (Herlihy et al., 2013). In the presence of the DPPIV inhibitors Diprotin A and DPPP 1c hydrochloride, the chemorepellent activity of DPPIV was significantly reduced, and suggesting that the protease activity of DPPIV mediates its ability to induce neutrophil chemorepulsion (Herlihy et al., 2013).

## DPPIV, the AprA-Like the Neutrophil Chemorepellent, Shows Efficacy in a Mouse Model of ARDS as Well as a Mouse Model of Rheumatoid Arthritis

A standard mouse model of ARDS is to damage the lungs with aspiration of a drug called bleomycin (Walters and Kleeberger, 2008). The mouse is lightly anesthetized, and 50 μl of saline with bleomycin is quickly pipetted through the mouth into the trachea as the mouse is inhaling. A gentle tailward shake of the mouse then disperses the bleomycin into the lungs. One day later, activated neutrophils start accumulating in the lungs (Walters and Kleeberger, 2008). Using this model, recombinant DPPIV or an equal volume of saline was introduced into the airspaces of the lungs of bleomycin-treated mice on day 1 and day 2 after bleomycin treatment by a similar aspiration procedure (Herlihy et al., 2013). Three days after bleomycin treatment, mice were euthanized. Phosphate buffered saline was put into the lungs through the trachea and then removed along with cells and other material in the airspaces in a procedure called bronchoalveolar lavage (BAL) (Herlihy et al., 2013). At day 3, the DPPIV treatments reduced both neutrophils in the BAL fluid as well as neutrophils remaining in the lungs (as determined by immunostaining of sections of the post-BAL lungs), but did not affect the numbers of macrophages and lymphocytes, suggesting that the effect of DPPIV is specific to neutrophils (Herlihy et al., 2013). Inhalation of DPPIV thus showed efficacy in a mouse model of ARDS.

Inflammatory arthritis, also known as rheumatoid arthritis (RA), is an autoimmune disorder with characteristic chronic inflammation due to neutrophil accumulation in the joints causing destruction of the joints (Kaplan, 2013). RA patients have lower level of DPPIV in plasma compared to non-inflammatory osteoarthritis (Busso et al., 2005). Injection of DPPIV directly into the joint in a mouse model of arthritis reduced the accumulation of neutrophils in the joint, and reduced the severity of arthritis and synovial inflammation (Herlihy et al., 2015). Together with the ARDS model results, this suggests that DPPIV can reduce inflammation by causing neutrophils to move away from a site of accumulation.

## Agonists of the DPPIV Receptor Par2 Induce Neutrophil Chemorepulsion and Show Efficacy in a Mouse Model of ARDS

As mentioned above, soluble DPPIV acts as a human and mouse neutrophil chemorepellent (Herlihy et al., 2013; White et al., 2018) via the activation of protease activated receptor 2 (PAR2) (White et al., 2018). PAR2 is a member of the PAR family of GPCRs, which consists of PAR1/2/3/4 (Ossovskaya and Bunnett, 2004). PAR2 is activated by the proteolytic cleavage of the extracellular N-terminus domain causing the remaining tethered N-terminus to act as a ligand that binds to extracellular loop 2 of the receptor thereby activating it (Vu et al., 1991; Gardell et al., 2008) DPPIV requires protease activated receptor (PAR2) to induce chemorepulsion of human neutrophils (White et al., 2018). Similar to DPPIV, the PAR2 agonists 2f-LIGRL-amide (a small peptide with modifications at the N and C termini), SLIGKVNH2 (a small peptide with a modifications at the C terminus), and AC55541 (a small molecule) induce chemorepulsion of human and mouse neutrophils (White et al., 2018). Although DPPIV induces stronger chemorepulsion of male neutrophils than female neutrophils, PAR2 agonists induce chemorepulsion of neutrophils from both male and female mice (White et al., 2018). In the mouse ARDS model described above, aspiration of SLIGKVNH2, starting 24 h after oropharyngeal aspiration of bleomycin, reduced neutrophil accumulation in lungs at day 3, similar to the effects of DPPIV described above (White et al., 2018), suggesting that aspiration of PAR2 agonists could be used to treat ARDS in human patients (Herlihy et al., 2013; White et al., 2018). Since DPPIV is a protease that cleaves many target molecules in addition to PAR2 (Zhu et al., 2003; Mulvihill and Drucker, 2014; Wronkowitz et al., 2014; White et al., 2018; Deacon, 2019; Trzaskalski et al., 2020), in terms of potential therapeutics, exogenous delivery of PAR2 agonists may have fewer side effects than exogenous delivery of DPPIV.

## Conclusion

Chemorepulsion is an essential process for development, morphogenesis, and immune responses in eukaryotes. The identification of AprA, an endogenous chemorepellent in *D. discoideum* led to the identification of DPPIV, a chemorepellent that acts on human (and mouse) neutrophils. This in turn led to the identification of small-molecule PAR2 agonists as neutrophil chemorepellents. Given by aspiration into the lungs, both DPPIV and a PAR2 agonist showed efficacy in a mouse model of ARDS. Although the activation of PAR2 can reduce damage caused by arthritis, ARDS, and ischemia (McLean et al., 2002; McCulloch et al., 2018; White et al., 2018), the activation of PAR2 can lead to unwanted effects such as fetal injury, fibrosis, and inflammatory, metabolic and cardiovascular disorders (Cicala, 2002; Redecha et al., 2008; Grimsey et al., 2011; Kagota et al., 2016; Shearer et al., 2016; Heuberger and Schuepbach, 2019). Although intriguing possibilities are that inhalation of a nebulized mist containing DPPIV and/or PAR2 agonists might be useful as therapeutics for ARDS, and localized delivery of these neutrophil chemorepellents might be useful in other neutrophil-driven diseases, caution will be needed to limit dosing to prevent systemic toxicity.

## Author Contributions

All authors listed have made a substantial, direct and intellectual contribution to the work, and approved it for publication.

## Conflict of Interest

The authors declare that the research was conducted in the absence of any commercial or financial relationships that could be construed as a potential conflict of interest.
